# Supplementary material for: Enhancement of β-Glucan Biological Activity Using a Modified Acid-Base Extraction Method from Saccharomyces cerevisiae
Source: Molecules. 2021 Apr 7;26(8):2113. doi: 10.3390/molecules26082113 (PMC8067753; doi:10.3390/molecules26082113)
Supplement: Supplementary file 1 [file molecules-26-02113-s001.pdf]

Supplementary Information

Enhancement of β-Glucan extraction from *Saccharomyces cerevisiae* and studying its promising properties

SI. 1. *β-glucan extraction methods*

SI.1.1 Acid-Base extraction method

SI.1.1.1 Autolysis of yeast cells

Autolysis of yeast cells was performed according to the reported method (Pengkumsri, Sivamaruthi et al. 2017). Typically, one hundred of the yeast cells were cultivated in 1 L of Yeast Extract-Glucose broth and incubated at 30 °C with agitation at 2 g for 48 h. Then, the yeast cells were collected via centrifugation at 6,530 × *g* for 10 min at 4 °C. The collected yeast cells (15 % w/v) were suspended in distilled water for 48 h at pH 5.0 and 50 °C with shaking at 2×*g*. After that, the autolysis reaction was terminated by incubating at 80 °C for 15 min in a water bath. Finally, the yeast cells were centrifuged at 4 °C and 2,800 × *g* for 10 min and the pellets were dried at 60 °C and stored at 4 °C until extraction

SI.1.1.2 Extraction of β-glucan

Acid and base combination (NaOH / CH_3COOH) was used in the extraction. Briefly, five folds of 1 M NaOH were mixed with the autolyzed yeast cells and incubated at 80 °C with stirring for 2 h. Then, the centrifugation at 6,000 × *g* for 25 min at 4 °C was done for collection of cell pellets before its suspension in three folds of distilled water, and re-centrifugation at 6,000 × *g* at 4 °C for 25 min. The cell pellets were dissolved in five folds of 1M CH_3COOH and incubated with stirring at 80 °C for 2 h. The pellets were then collected by centrifugation at 6,000 × *g* for 25 min at 4 °C. The obtained pellets were washed 3 times with water and dried in oven at 60 °C. The extracted sample was coded to S1.

SI.1.2 Water extraction method

SI.1.2.1 Autolysis of yeast cells

The method of autolysis was based on the documented by Piotrowska and Masek with some modifications (Piotrowska and Masek 2015). Briefly, 100 g of yeast cells were added to 50 mL of 10 mM phosphate buffer (pH 8.0), then the mixture was subjected to 5 min of sonication instead of glass beads shaking for 4h. The next step was centrifugation (5000 × *g*, 15 min) and washing twice with a phosphate buffer. In order to separate protein substances, 1% of sodium dodecyl sulphate (SDS) was added to the remainder, followed by shaking for 3h at 30 °C. The resulting cell walls preparation was dried in absolute ethanol**.**

SI.1.2.2 Extraction of β-glucan

The output from the previous step was transferred to a suitable amount of water and autoclaved for 4h according to the proposed method (Freimund, Sauter et al. 2003, Liu, Wang et al. 2008). The insoluble fraction of water was then centrifuged at 6,000 × *g* for 25 min under cooling, and the collected pellets were air dried at room temperature. The code of this extracted sample was S2.

SI.2. Characterization

SI.2.1 Fourier Transform infrared spectroscopy (FTIR)

Functional groups of β-Glucan extracted (S1 and S2) were determined using FTIR spectrometer (Nicolet Nexus 470 FTIR instrument, USA) with standard KBr method (Kesika, Prasanth et al. 2015, Sivamaruthi, Prasanth et al. 2015), The spectral range was 4000 to 450 cm^{-1} at 4 cm^{-1} resolution. All measurements were acquired in duplicate normalization. Spectra were pre-processed (baseline correction, normalization, and second derivative) with OMNIC 32 software before analysis.

SI.2.2. High performance liquid chromatography (HPLC)

Protein and carbohydrate contents were analyzed using HPLC using the published methods (Dubois, Gilles et al. 1956, Bradford 1976). HPLC separation was carried out using Luna 5u C18 column (250 x 4.6 mm). The mobile phase used was acetonitrile: water (1:1 v/v) and the flow rate was 0.5 mL/min. The injection volume of both sample and standard were 20 µL and the pH was 5. The detection of β-Glucan was taken place at 305 nm.

SI.2.3. Optical confirmation

A double-beam spectrophotometer (Shimadzu UV-2101 combined with PC) was used to record transmittance (T) and reflectance (R) at room temperature (Zaki, Abd-Elrahman et al. 2018).

SI.3. Aflatoxins removal

The efficiency of the extracted β-glucan to remove aflatoxins (AFS) was tested using batch mode of experiment. Serial concentrations of the extracted β-glucan, acid-base and water extraction, (0.1, 0.2, 0.3, 0.4, 0.5 mg of dry weight) were tested for its ability to remove aflatoxins (1000 ng/mL). The extracted β-glucan samples were dissolved in 0.1 M phosphate buffered saline (PBS) pH 6.2 and then AFS were added. The mixture was incubated with agitation (4 × *g*) at 30 °C for 24 h. To determine the amount of the residual AFS in the supernatant after 24 h, the samples were centrifuged at 7000× *g* for 10 min and the residual AFS was determined using TLC method. The control sample consisted of the same mixtures, but without β-glucan.

Thin layer chromatography (TLC) was used for AFS analysis. Briefly, 10 µL of the above residual was spotted on TLC along with the control. The running solvent were: Chloroform: methyl alcohol (97: 3 v/v). Aflatoxins B_1 detected as bright blue fluoresces at R_f 0.78 under long waves UV light (Zohri, Aboul-Nasr et al. 2017). Moreover, the efficiency of β-glucan was estimated by the determination of AFS concentration at the beginning and the end of the experiments (Piotrowska and Masek 2015).

SI.4. Antimicrobial activity

SI.4.1. Isolation of Microorganisms

All bacterial isolates were kindly provided from the department of Medical Microbiology and Immunology, Faculty of Medicine, Assiut University, Assiut, Egypt. These clinical isolates were obtained from clinical cases of infections admitted to Assiut University hospital, Assiut, Egypt, as diarrhea, urinary tract infections, keratitis, bacterial and fungal pneumonia, otomycosis, and thrush and wound infections. The clinical isolates were proved by using the VITEK 2 automated microbiology system (BioMérieux). Fungal strains were isolated in our lab during the PhD study of Dr. Enas M. Amer. Yeast isolate has been kindly provided by Dr. Mohamed Farhan from his PhD study (Farhan, Moharram et al. 2019).

The clinical bacterial isolates used were multidrug resistant strains, they were resistant to β-lactam (amoxicillin and oxacillin), cephalosporins (cefazolin, ceftriaxone and cefepime) and macrolides (erythromycin and clarithromycin). They included Gram positive bacteria such as *Staphylococcus aureus (S. aureus*), Methicillin-resistant *Staphylococcus aureus* (MRSA), *Streptococcus pneumoniae (S. pneumoniae),* and Gram negative bacteria such as *Escherichia coli (E. coli), Klebsiella pneumoniae (K. pneumoniae)* and *Pseudomonas aeruginosa (P. aeruginosa)*. The tested fungal strains are *Aspergillus flavus (A. flavus EN1 KY609), A. niger (A. niger EN2 KY6095)* and *Candida albicans (C. albicans* MH 534906*).*

SI.4.2. Initial evaluation of the β -glucan antibacterial and antifungal activities

The antimicrobial activities of the S1 and S2 β-glucan extracted were evaluated by agar well diffusion assay (Ginovyan, Keryan et al. 2015). Suspensions of the tested microbial strains were adjusted to turbidity of 0.5 McFarland Standard, which equals to 1-2 $x10^6$ CFU /mL for bacteria and 1-5 $x10^5$ CFU /mL for fungi. Typically, 100 µL of the tested β-glucans, S1 and S2, with concentration of

100 mg/mL and controls (100 mg/mL) were dispensed into the wells. Vancomycin (50 µg/ mL), gentamicin (10 µg/ mL) and fluconazole (25 µg /mL) as positive control were used for Gram positive bacteria, Gram negative bacteria, and fungi, respectively. The negative control agent was 1% DMSO and water. The plates were incubated for 24 h at 37 °C for bacteria and *C. albicans* at 28°C for *A. flavus* and *A. niger*. The diameters of inhibition zones (ZOI) around the wells were measured in mm. All experiments were independently repeated three times and the obtained data were statistically evaluated using GraphPad prism 5.03 software (GraphPad Software, Inc.; USA).

SI.4.3. Determination of MIC values for the extracted β-glucan

Minimum inhibitory concentrations (MIC) of β-glucans (S1 and S2) were determined using broth microdilution method (Wiegand, Hilpert et al. 2008). For this assay, the positive control agents were vancomycin (0.7-50 µg /mL), and gentamicin (0.15- 10 µg /mL), while the negative control was DMSO (1 %).

SI.5. Immunomodulatory effects

SI.5.1. Cell culture

Human monocytes cells (HMCs) were maintained in RPMI media containing HEPES (10 mM), and FBS (10 %) at cell concentration of lower than 10^6 cells/mL. β-glucans were added at the cell's concentration of 10 µg/mL for 16 h. A biological dye, phorbol myristate acetate (PMA), was used as a positive control.

SI.5.2. Cytokine Assays

Supernatants were collected from the treated cells. Naïve cells at the same numbers were used as negative control. IL-2, IL-6, TNFα and IFN-ɤ were measured by ELISA (R&D kits, USA) which is agreeing to the manufacturer's guidelines. The levels of these cytokines were calculated from the

standard curves generated using the provided recombinant proteins according the manufacturer's instructions.

SI.6. Anticancer properties

SI.6.1. Tissue culture

WI-38 human normal lung fibroblast cells, adenocarcinomic human alveolar basal epithelial cells, MDA-MB-231 breast carcinoma cells, and HepG-2 hepatocellular carcinoma cells were obtained from VACSERA, Cell Culture Unit, Cairo, Egypt. All cell lines were first purchased from the American Tissue Culture Collection (ATCC). The WI-38, A549, MDA-MB-23, and HePG-2 cells were grown in RPMI-1640 medium supplemented with 10% inactivate fetal bovine serum (FBS) and 1% penicillin/streptomycin.

SI.6.2. Cell proliferation assay

Cell proliferation was measured with MTT (3-[4, 5-dimethyl-2-thiazolyl]-2, 5- diphenyl-2H-tetrazolium bromide). The principles of MTT mainly depend on the conversion of the soluble MTT form to non-soluble form (tetrazolium salt to formazan) in the presence of succinate-tetrazolium reductase enzyme in the mitochondria of living cells, the formation of later form is proportional to the number of living cells and can be measured colometry at wavelength of 570 nm. Briefly, cells were cultured at initial concentration of $1x10^4$ cell/ well in 96-well plates and 24 h. Later, cells were treated with β-glycan (0.0, 0.0, 62.5, 125, 250, 500 and 1000 μg/mL) for 24 h. Next, 10 μL/ well of MTT solution (5 mg/mL) was added and incubated for 4 h and then intracellular formazan dye resulting from the metabolically active cells can be solubilized and quantified by measuring its absorbance at 570 nm using ELISA reader.

SI.6.3. Apoptosis and necrosis using FACS analysis

The apoptotic and necrotic cell death induced by water extracted β-glycan were measured using Annexin V and propidium iodide (PI). In brief, WI-38, MDA-MB-23, and HePG-2 cells were cultured in 10 tissue culture dishes with initial number of 4 x 10^5 cell/ Ml in RPMI growth media and treated with (0.0 and 250 µg/mL) of β-glycan and incubated for 24 h. Briefly, the cells washed once with PBS and suspended in 100 µL of 1X Annexin V as binding buffer and add 5µL FITC and incubated for 15 minutes at room temperature then 5 µL of PI were added to each tube. Finally, 400 µL of 1X Annexin V were added to each tube and analyzed using Becton Dickinson FACS.

SI.6.4. Data analysis

All experiments were repeated three times and the obtained data were expressed as the mean ± SD and compared using a two-tailed student's t-test. Results were analyzed using GraphPad Prism 5.03 software (GraphPad Software, Inc.; USA) and considered significant if p values were less than 0.05.

References

Bradford, M. M. (1976). "A rapid and sensitive method for the quantitation of microgram quantities of protein utilizing the principle of protein-dye binding." Analytical biochemistry **72**(1-2): 248-254.

Dubois, M., K. A. Gilles, J. K. Hamilton, P. t. Rebers and F. Smith (1956). "Colorimetric method for determination of sugars and related substances." Analytical chemistry **28**(3): 350-356.

Farhan, M. A., A. M. Moharram, T. Salah and O. M. Shaaban (2019). "Types of yeasts that cause vulvovaginal candidiasis in chronic users of corticosteroids." Medical mycology **57**(6): 681-687.

Freimund, S., M. Sauter, O. Käppeli and H. Dutler (2003). "A new non-degrading isolation process for 1, 3-β-D-glucan of high purity from baker's yeast Saccharomyces cerevisiae." Carbohydrate Polymers **54**(2): 159-171.

Ginovyan, M., A. Keryan, I. Bazukyan, P. Ghazaryan and A. Trchounian (2015). "The large scale antibacterial, antifungal and anti-phage efficiency of Petamcin-A: new multicomponent preparation for skin diseases treatment." Annals of clinical microbiology and antimicrobials **14**(1): 28.

Kesika, P., M. I. Prasanth and K. Balamurugan (2015). "Modulation of Caenorhabditis elegans immune response and modification of Shigella endotoxin upon interaction." Journal of basic microbiology **55**(4): 432-450.

Liu, X.-Y., Q. Wang, S. W. Cui and H.-Z. Liu (2008). "A new isolation method of β-D-glucans from spent yeast Saccharomyces cerevisiae." Food Hydrocolloids **22**(2): 239-247.

Pengkumsri, N., B. S. Sivamaruthi, S. Sirilun, S. Peerajan, P. Kesika, K. Chaiyasut and C. Chaiyasut (2017). "Extraction of β-glucan from Saccharomyces cerevisiae: comparison of different extraction methods and in vivo assessment of immunomodulatory effect in mice." Food Science and Technology **37**(1): 124-130.

Piotrowska, M. and A. Masek (2015). "Saccharomyces cerevisiae cell wall components as tools for ochratoxin A decontamination." Toxins **7**(4): 1151-1162.

Sivamaruthi, B. S., M. I. Prasanth and K. Balamurugan (2015). "Alterations in Caenorhabditis elegans and Cronobacter sakazakii lipopolysaccharide during interaction." Archives of microbiology **197**(2): 327-337.

Wiegand, I., K. Hilpert and R. E. Hancock (2008). "Agar and broth dilution methods to determine the minimal inhibitory concentration (MIC) of antimicrobial substances." Nature protocols **3**(2): 163.

Zaki, S. A., M. Abd-Elrahman, A. Abu-Sehly, N. Shaalan and M. Hafiz (2018). "Thermal annealing of SnS thin film induced mixed tin sulfide oxides-Sn2S3 for gas sensing: Optical and electrical properties." Materials Science in Semiconductor Processing **75**: 214-220.

Zohri, A., M. Aboul-Nasr, M. Adam, M. Mustafa and E. Amer (2017). "Impact of enzymes and toxins potentiality of four aspergillus species to cause aspergillosis." Biol Med (Aligarh) **9**(409): 2.
